# Predominance of Gram-Negative Pathogens and Treatment Complexity in Peritoneal Dialysis-Associated Peritonitis: A Single-Center Experience

**DOI:** 10.3390/life16040558

**Published:** 2026-03-29

**Authors:** Daniela Marinescu, Laurențiu Augustus Barbu, Tiberiu-Ștefăniță Țenea-Cojan, Daniela-Teodora Maria, Sorin-Ioan Zaharie, Răzvan Alexandru Marinescu, Valeriu Șurlin, Ana-Maria Ciurea, Anca-Elena Duduveche

**Affiliations:** 1 Department of Surgery, Emergency County Hospital, University of Medicine and Pharmacy of Craiova, 2 Petru Rares Street, 200349 Craiova, Romania; dtmarinescu@yahoo.com; 2 Department of Surgery, Railway Clinical Hospital Craiova, University of Medicine and Pharmacy of Craiova, 2 Petru Rares Street, 200349 Craiova, Romania; tiberiu.tenea@umfcv.ro; 3 Department of Nephrology, Emergency County Hospital, University of Medicine and Pharmacy of Craiova, 2 Petru Rares Street, 200349 Craiova, Romania; daniela.maria@umfcv.ro (D.-T.M.); szaharie3@yahoo.com (S.-I.Z.); 4 Department of Plastic Surgery, University of Medicine and Pharmacy of Craiova, 200349 Craiova, Romania; razvanalexandrumarinescu98@gmail.com; 5 Academy of Romanian Scientists, 3 Ilfov Street, 050044 Bucharest, Romania; 6 Department of Oncology, Emergency County Hospital, University of Medicine and Pharmacy of Craiova, 2 Petru Rares Street, 200349 Craiova, Romania; amciurea14@gmail.com; 7 Department of Infectious Diseases, Faculty of Medicine, University of Medicine and Pharmacy of Craiova, 200349 Craiova, Romania; anca.duduveche@umfcv.ro

**Keywords:** peritoneal dialysis, peritoneal dialysis-associated peritonitis, Gram-negative bacteria, antimicrobial therapy, relapsing peritonitis, treatment complexity, single-center study

## Abstract

**Background:** Peritoneal dialysis-associated peritonitis (PDAP) remains a major complication of peritoneal dialysis and an important cause of technique failure. Increasing evidence suggests marked inter-center variability in PDAP microbiology, with a growing contribution of Gram-negative pathogens in some settings. **Methods:** We performed a single-center, retrospective observational study of adult peritoneal dialysis patients with PDAP treated between January 2020 and December 2024. Episodes were defined according to International Society for Peritoneal Dialysis criteria. Clinical, microbiological, and antimicrobial treatment data were analyzed, with particular focus on relapsing peritonitis and treatment complexity. **Results:** Thirty-three patients were included (median age 59 years; 51.5% male). Gram-negative organisms were the most frequent causative agents (48.5%), followed by Gram-positive bacteria (21.2%), fungal pathogens (6.1%), and culture-negative episodes (6.1%). Relapsing or recurrent peritonitis occurred in 12.1% of cases and was significantly associated with increased antimicrobial treatment complexity, with all relapsing episodes requiring three or more antimicrobial agents (*p* = 0.02). **Conclusions:** Gram-negative pathogens predominated in this single-center PDAP cohort and showed trends toward greater antimicrobial treatment complexity and a higher burden of relapsing episodes; however, these findings should be interpreted cautiously due to the limited sample size and lack of statistical significance. These findings nevertheless support the need for center-specific microbiological surveillance and individualized management strategies in peritoneal dialysis-associated peritonitis.

## 1. Introduction

Peritoneal dialysis (PD) is a widely used renal replacement therapy for patients with end-stage kidney disease, offering several advantages over hemodialysis, including preservation of residual renal function, improved hemodynamic stability, and enhanced quality of life due to its home-based nature [1]. Despite these benefits, the long-term success of PD is limited by infectious complications, among which peritoneal dialysis-associated peritonitis (PDAP) remains a major cause of morbidity, technique failure, transfer to hemodialysis, and mortality [2].

Over recent decades, international efforts have focused on reducing the incidence and improving the management of PD-associated peritonitis. The International Society for Peritoneal Dialysis (ISPD) regularly updates evidence-based recommendations addressing prevention, diagnosis, and treatment, emphasizing the importance of microbiological surveillance and awareness of center-specific epidemiology to guide empirical antimicrobial therapy [2,3]. Current guidelines recommend empirical dual coverage against Gram-positive and Gram-negative organisms, with subsequent adjustment based on culture results and local resistance patterns.

Historically, Gram-positive bacteria—particularly coagulase-negative staphylococci and *Staphylococcus aureus*—have been reported as the predominant causative agents of PD-associated peritonitis in many cohorts [4,5]. However, growing evidence indicates substantial inter-center and geographic variability in the microbiological spectrum of PDAP, with several studies reporting an increasing contribution of Gram-negative and enteric organisms [6,7]. These differences are clinically relevant, as Gram-negative and polymicrobial infections have been associated with a more severe disease course and increased treatment complexity [8].

In addition to microbiological factors, patient- and treatment-related characteristics—including diabetes mellitus, anemia, nutritional status, dialysis modality, and the need for assisted PD—have been associated with an increased risk of PD-associated peritonitis and adverse outcomes [9,10]. Relapsing or recurrent peritonitis represents a clinically meaningful entity, often reflecting persistent infection or host vulnerability, and has been linked to increased antimicrobial exposure and technique failure [11].

Culture-negative peritonitis remains an important quality indicator in PD programs, reflecting sampling technique and microbiological processing, and continues to pose diagnostic and therapeutic challenges [7,12]. Together with variability in training practices and center-level organization, these factors support the relevance of center-specific analyses [13].

Although large registry-based and multicenter studies provide important epidemiological insights, single-center observational studies remain valuable for capturing real-world practice, local microbiological profiles, and antimicrobial treatment strategies, particularly in centers with limited published data [14].

Despite the growing body of literature on peritoneal dialysis-associated peritonitis, data from smaller peritoneal dialysis centers in Central and Eastern Europe remain limited. In such settings, local microbiological patterns, patient characteristics, and treatment practices may differ from those reported in large registry-based or multicenter studies. Single-center analyses from these regions may therefore provide valuable insights into real-world practice and inform center-specific preventive and therapeutic strategies.

Therefore, the aim of the present study was to describe the microbiological findings, clinical characteristics, and antimicrobial treatment patterns of peritoneal dialysis-associated peritonitis in a single-center cohort, with particular attention to pathogen distribution, treatment complexity, and factors associated with relapsing peritonitis.

## 2. Materials and Methods

### 2.1. Study Design and Setting

We conducted a single-center, observational study evaluating peritoneal dialysis-associated peritonitis (PDAP) in patients treated with chronic peritoneal dialysis (PD) at the Department of Nephrology, Emergency County Clinical Hospital (Spitalul Clinic Județean de Urgență) Craiova, Romania. The study period spanned 1 January 2020 to 31 December 2024. The study aimed to describe the microbiological spectrum, clinical characteristics, and antimicrobial treatment patterns of PDAP, with a particular focus on factors associated with relapsing/recurrent episodes and treatment complexity.

### 2.2. Study Population and Eligibility Criteria

We screened all adult patients (≥18 years) receiving PD who experienced at least one episode of suspected peritonitis during the study period. Episodes were included if they fulfilled the diagnostic definition for PDAP based on International Society for Peritoneal Dialysis (ISPD) criteria, requiring at least two of the following: (i) clinical features consistent with peritonitis (e.g., abdominal pain and/or cloudy effluent); (ii) dialysis effluent white blood cell count >100/µL with >50% polymorphonuclear leukocytes; and (iii) positive dialysis effluent culture.

Episodes were excluded if peritonitis was deemed non-infectious, if key diagnostic data were missing (e.g., absent effluent cell count and unavailable culture results), or if peritonitis occurred in the setting of an abdominal surgical catastrophe where PDAP could not be reliably adjudicated.

### 2.3. Definitions of Peritonitis Episodes

Episodes were classified according to ISPD terminology:

Culture-negative peritonitis: peritonitis fulfilling clinical and effluent cell count criteria with negative cultures after standard incubation/processing.

Relapsing peritonitis: an episode occurring within 4 weeks of completion of therapy for a prior episode caused by the same organism (or following a sterile episode), consistent with ISPD definitions.

Recurrent peritonitis: an episode occurring within 4 weeks of completion of therapy but caused by a different organism. For analyses, relapsing and recurrent episodes were grouped when appropriate (relapsing/recurrent peritonitis), consistent with the clinical aim of identifying “repeat-episode” vulnerability. In addition, relapsing peritonitis was analyzed separately due to its distinct clinical implications and potential association with treatment complexity. The unit of analysis for microbiological and treatment-related variables was the peritonitis episode. When multiple episodes occurred in the same patient, each episode was analyzed separately. Patient-level characteristics were analyzed at baseline.

### 2.4. Data Collection and Variables

Data were extracted retrospectively from electronic and paper medical records using a predefined collection template. Collected variables included:

Demographics: age, sex, residence (urban/rural).

Comorbidities and clinical context: diabetes mellitus, hypertension, evidence of hyperglycemia/increased glucose absorption, defined as documented elevated blood glucose levels or increased peritoneal glucose absorption recorded in the clinical record during the peritonitis episode, and other clinically relevant comorbidities when available.

Laboratory parameters (at presentation): hemoglobin and serum sodium (hyponatremia defined as <135 mmol/L).

Microbiology: effluent culture result and organism identification, categorized as Gram-negative, Gram-positive, fungal, other isolates, or culture-negative. The category “other isolates” included less frequent or uncommon microorganisms, as well as occasional polymicrobial infections, each identified in a small number of cases. Blood culture results were recorded when performed.

Antimicrobial therapy: empirical regimen and subsequent changes, number of antimicrobial agents administered per episode/patient, and major antimicrobial classes used.

Key study outcomes: (i) distribution of causative microorganisms; (ii) frequency of relapsing/recurrent peritonitis; and (iii) antimicrobial treatment complexity, operationalized as the use of ≥3 antimicrobial agents during management.

### 2.5. Microbiological Sampling and Processing

Peritoneal effluent was collected aseptically prior to antibiotic administration whenever feasible. Samples were transported promptly to the microbiology laboratory according to local procedures. Culture processing and organism identification were performed using standard clinical microbiology methods in routine practice. Culture-negative peritonitis was defined as the absence of growth despite standard diagnostic evaluation and processing. When obtained, blood cultures were interpreted using routine institutional standards.

### 2.6. Antimicrobial Management

Empirical antibiotic therapy was initiated promptly after sampling, following contemporary ISPD recommendations and local protocol, with coverage directed against both Gram-positive and Gram-negative organisms. Antimicrobial therapy was subsequently adjusted based on culture results, clinical response, and antimicrobial susceptibility patterns when available. Treatment complexity was quantified by the total number of antimicrobial agents used during the treatment course.

### 2.7. Statistical Analysis

Continuous variables were assessed for distribution and are presented as median (interquartile range, IQR). Categorical variables are presented as counts and percentages. Comparisons between Gram-negative and Gram-positive episodes were performed using the Mann–Whitney U test for continuous variables and Fisher’s exact test for categorical variables, as appropriate. A two-sided *p* value < 0.05 was considered statistically significant. Statistical analyses were performed using IBM SPSS Statistics (version 26.0; IBM Corp., Armonk, NY, USA).

### 2.8. Ethics

The study was conducted in accordance with the Declaration of Helsinki and was approved by the Ethics Committee of the Emergency County Clinical Hospital of Craiova (approval number 7914/16 February 2026). Data extraction and statistical analysis were performed only after ethics committee approval was obtained. Written informed consent for the use of anonymized medical data for scientific purposes was obtained from all patients at the time of hospital admission, in accordance with institutional regulations.

## 3. Results

The study included 33 patients, accounting for a total of 33 peritonitis episodes. The median age was 59 years (IQR 44–74), with 51.5% males. Diabetes mellitus and hypertension were present in 36.4% and 60.6% of patients, respectively. Median hemoglobin was 11.1 g/dL (IQR 10.3–12.1), and 54.5% of patients showed hyperglycemia or increased glucose absorption (Table 1). Relapsing or recurrent peritonitis occurred in 12.1% of cases.

Gram-negative organisms were the most frequent etiological agents (48.5%), followed by Gram-positive bacteria (21.2%), fungal pathogens (6.1%), and culture-negative episodes (6.1%) (Table 1). The most common Gram-negative isolates were *Escherichia coli* (24.2%) and *Enterobacter* spp. (15.2%); a full distribution of all identified microorganisms is presented in Table 2.

The main isolated microorganisms are summarized in Table 2. Less frequent isolates were identified in a small number of cases. These cases correspond to less frequent or uncommon microorganisms and occasional polymicrobial infections, which were not analyzed as a separate category.

Culture-negative peritonitis accounted for 6.1% of cases (2 of 33). Blood cultures were negative in all culture-negative cases.

When stratified by Gram status, no statistically significant differences were observed between groups; however, Gram-negative peritonitis episodes showed numerically lower hemoglobin levels, higher rates of hyponatremia and relapse, and more frequent use of ≥3 antimicrobial agents, suggesting a trend toward a more severe clinical profile (Table 3).

Relapsing or recurrent peritonitis was identified in four patients (12.1%), while relapsing episodes were also analyzed separately due to their clinical relevance (Table 4). Although no statistically significant differences were observed, relapse occurred more frequently in patients with diabetes, lower hemoglobin levels, and Gram-negative etiology. Most relapsing episodes were associated with Gram-negative organisms, particularly *Escherichia coli* and *Enterobacter* spp., consistent with the overall microbiological distribution of the cohort.

Most patients received combination antimicrobial therapy, with a median of three agents per patient (IQR 2–4). Cephalosporins and aminoglycosides were the most frequently used drug classes, and empirical dual coverage for Gram-positive and Gram-negative organisms was implemented in the majority of cases (90.9%), consistent with the observed microbiological profile (Table 5).

Relapsing peritonitis was observed in four patients (12.1%) (Table 6). Among the analyzed variables, the use of ≥3 antimicrobial agents was significantly more frequent in relapsing episodes, while no statistically significant differences were identified for demographic, clinical, or microbiological characteristics, although some variables showed non-significant trends.

## 4. Discussion

In this single-center observational study, we characterized the microbiological spectrum, clinical features, and antimicrobial treatment patterns of peritoneal dialysis-associated peritonitis (PDAP) in a contemporary cohort of peritoneal dialysis (PD) patients. The key findings were a predominance of Gram-negative pathogens, a relatively low proportion of culture-negative episodes, and a significant association between relapsing peritonitis and increased antimicrobial treatment complexity. Together, these findings emphasize the importance of center-specific epidemiology and individualized management strategies in PDAP.

### 4.1. Microbiological Profile in the Context of Contemporary PD Practice

Historically, Gram-positive organisms—particularly coagulase-negative staphylococci—have been reported as the most frequent causative agents of peritoneal dialysis-associated peritonitis in large registry-based studies [15,16,17]. These infections are generally associated with more favorable clinical outcomes compared with Gram-negative or fungal peritonitis.

In contrast, our cohort was characterized by a predominance of Gram-negative pathogens, accounting for nearly half of all episodes. This finding highlights the importance of local microbiological epidemiology when selecting empirical antimicrobial therapy and reflects known inter-center variability influenced by patient characteristics and local practice patterns [17].

Similar heterogeneity has been described in single-center studies, including cohorts from Italy and China, where Gram-negative peritonitis has been associated with worse clinical outcomes and increased risk of treatment failure [18,19,20,21].

Among Gram-negative pathogens, *Escherichia coli* and *Enterobacter* spp. were the most frequently isolated organisms in our study. In line with previous reports, Gram-negative peritonitis episodes in our cohort showed numerically lower hemoglobin levels, higher rates of hyponatremia, and more frequent relapse, suggesting a trend toward greater clinical severity; however, these differences did not reach statistical significance and should be interpreted cautiously [22,23,24].

Relapsing peritonitis remains a clinically relevant complication, often associated with increased treatment burden and poten-tial technique failure. In our cohort, the association between relapse and the use of multiple antimicrobial agents likely reflects greater disease complexity rather than treatment choice alone, supporting the role of treatment intensity as a surrogate marker of severity [25,26].

### 4.2. Culture-Negative Peritonitis

The proportion of culture-negative peritonitis in our cohort was relatively low (6.1%) compared with earlier reports. Culture-negative episodes are widely regarded as a quality indicator in peritoneal dialysis programs, reflecting the adequacy of sampling technique, transport conditions, and microbiological processing. Current International Society for Peritoneal Dialysis (ISPD) guidelines recommend that culture-negative episodes should account for less than 15–20% of all peritonitis cases [2,9,27,28]. In this context, the low rate observed in our study suggests appropriate adherence to recommended diagnostic practices and supports the reliability of the reported microbiological findings.

### 4.3. Relapsing Peritonitis and Systemic Vulnerability

Relapsing or recurrent peritonitis occurred in 12.1% of patients, a frequency comparable to that reported in registry-based and single-center cohorts. While relapsing and recurrent episodes were grouped for overall analysis, relapsing peritonitis was evaluated separately due to its stronger association with treatment complexity. Although statistical significance was not reached, relapse was more frequent among patients with diabetes mellitus, lower hemoglobin levels, and Gram-negative etiology, consistent with previously described high-risk profiles [4,29].

Lower hemoglobin levels observed in relapsing cases may reflect chronic inflammation or reduced physiological reserve. Experimental studies suggest that PD-associated peritonitis induces systemic inflammatory responses that may contribute to anemia and impaired host defense mechanisms [2,30,31].

In our cohort, relapsing peritonitis was significantly associated with increased antimicrobial treatment complexity, with all relapsing episodes requiring three or more antimicrobial agents. This likely reflects greater clinical and microbiological complexity rather than treatment choice alone, consistent with previous studies linking relapse to higher microbial burden, delayed therapeutic response, and biofilm-related infection [2,32].

Most patients received combination antimicrobial therapy with empirical dual Gram-positive and Gram-negative coverage, in line with ISPD recommendations [2,33,34,35,36]. In this context, the association between treatment complexity and relapse supports the interpretation of antimicrobial burden as a surrogate marker of disease severity [37,38].

Finally, the identification of high-risk patient profiles, including those with diabetes, anemia, or prior Gram-negative peritonitis, may support targeted follow-up strategies and individualized management approaches. Integration of local epidemiological data into antimicrobial stewardship and patient management may improve outcomes and reduce the risk of relapse.

### 4.4. Strengths and Limitations

The main strength of this study lies in its detailed, real-world characterization of peritoneal dialysis-associated peritonitis in a single-center cohort with a distinct microbiological and therapeutic profile. Unlike large registry-based analyses, which often prioritize incidence and broad outcomes, our study provides granular clinical, laboratory, and treatment-level data, allowing a nuanced description of pathogen distribution, antimicrobial use, and factors associated with relapsing peritonitis in routine clinical practice.

Several limitations should be acknowledged. First, the relatively small sample size and single-center, observational design limit statistical power and preclude causal inference. As a consequence, several clinically relevant associations—such as the higher frequency of relapse, anemia, hyponatremia, and increased antimicrobial use in Gram-negative peritonitis—did not reach statistical significance and should be interpreted as hypothesis-generating signals rather than definitive conclusions. Second, we were unable to systematically evaluate the impact of peritoneal dialysis modality (CAPD versus APD), catheter characteristics, exit-site status, or detailed patient training and retraining practices, all of which have been shown to influence peritonitis risk and outcomes in larger cohorts. Similarly, markers of nutritional status such as serum albumin were not consistently available and could not be included in multivariable analyses.

Despite these limitations, the relatively small size of our cohort reflects the reality of a medium-sized peritoneal dialysis center and represents an important strength in terms of external validity for similar settings. Single-center studies remain particularly valuable for capturing local microbiological epidemiology, antimicrobial prescribing patterns, and treatment complexity that may be underrepresented in large registries. In this context, our findings highlight a predominance of Gram-negative pathogens, a low proportion of culture-negative episodes consistent with quality benchmarks, and a strong association between relapsing peritonitis and increased antimicrobial treatment complexity.

Additionally, the definition of treatment complexity as the use of three or more antimicrobial agents may reflect local prescribing patterns and escalation practices rather than infection severity alone. Therefore, this measure should be interpreted cautiously and may not fully capture underlying disease severity.

Taken together, this study provides locally relevant, real-world evidence that complements existing registry data and supports the need for center-specific microbiological surveillance and individualized management strategies. Although confirmatory multicenter studies are needed, our results contribute meaningful insights into the clinical phenotype and therapeutic burden of peritoneal dialysis-associated peritonitis in contemporary practice.

## 5. Conclusions

In this single-center observational study, Gram-negative organisms emerged as the predominant causative agents of peritoneal dialysis-associated peritonitis and showed trends toward greater treatment complexity and a higher frequency of relapsing episodes; however, these findings should be interpreted cautiously due to the limited sample size and lack of statistical significance.

Despite these limitations, our results highlight the clinical relevance of local microbiological epidemiology and patient vulnerability factors in shaping peritonitis outcomes. These findings support the need for regular, center-specific microbiological surveillance and individualized risk stratification in peritoneal dialysis practice to optimize empirical antimicrobial strategies and improve patient management.

## Figures and Tables

**Table 1:** Baseline demographic, clinical and microbiological characteristics.

Characteristic	Total (n = 33)
**Age, years, median (IQR)**	59 (44–74)
**Male sex, n (%)**	17 (51.5%)
**Rural residence, n (%)**	11 (33.3%)
**Diabetes mellitus, n (%)**	12 (36.4%)
**Hypertension, n (%)**	20 (60.6%)
**Hemoglobin, g/dL, median (IQR)**	11.1 (10.3–12.1)
**Hyperglycemia/increased glucose absorption, n (%)**	18 (54.5%)
**Relapsing/recurrent peritonitis, n (%)**	4 (12.1%)
**Microbiological etiology**	
**– Gram-negative bacteria**	16 (48.5%)
**– Gram-positive bacteria**	7 (21.2%)
**– Fungal**	2 (6.1%)
**– Culture-negative**	2 (6.1%)

**Table 2:** Main isolated microorganisms in PDAP episodes.

Microorganism	N	%
*Escherichia coli*	8	25.8
*Enterobacter* spp.	5	16.1
*Staphylococcus* spp.	6	19.4
*Klebsiella* spp.	2	6.5
*Candida albicans*	2	6.5
*Pseudomonas* spp.	1	3.2
*Streptococcus* spp.	1	3.2
*Proteus* spp.	1	3.2

**Table 3:** Clinical, laboratory, and treatment characteristics according to Gram status.

Variable	Gram-Negative (n = 16)	Gram-Positive (n = 7)	*p*-Value
**Age, years, median [IQR]**	61 (48–75)	55 (41–70)	0.61 ^1^
**Female sex, n (%)**	7 (43.8)	4 (57.1)	0.68 ^2^
**Diabetes mellitus, n (%)**	7 (43.8)	2 (28.6)	0.67 ^2^
**Hemoglobin, g/dL, median [IQR]**	10.8 [10.1–11.9]	11.6 [10.9–12.5]	0.19 ^1^
**Hyponatremia (<135 mmol/L), n (%)**	9 (56.3)	3 (42.9)	0.69 ^2^
**Relapsing peritonitis, n (%)**	3 (18.8)	0 (0.0)	0.53 ^2^
**≥3 antibiotics used, n (%)**	11 (68.8)	3 (42.9)	0.36 ^2^

Note: Data are presented as median [IQR] or n (%). ^1^ Mann–Whitney U test; ^2^ Fisher’s exact test. IQR: interquartile range.

**Table 4:** Factors associated with relapsing/recurrent peritonitis.

Variable	Relapse (n = 4)	No Relapse (n = 29)	*p*-Value
**Age, years, median (IQR)**	66 (58–77)	57 (43–72)	0.42 ^1^
**Male sex, n (%)**	3 (75.0%)	14 (48.3%)	0.61 ^2^
**Diabetes mellitus, n (%)**	3 (75.0%)	9 (31.0%)	0.12 ^2^
**Hemoglobin, g/dL, median (IQR)**	10.2 (9.6–11.0)	11.3 (10.5–12.2)	0.08 ^1^
**Gram-negative etiology, n (%)**	3 (75.0%)	13 (44.8%)	0.34 ^2^

Note: Data are presented as median (IQR) or n (%). ^1^ Mann–Whitney U test; ^2^ Fisher’s exact test. IQR: interquartile range.

**Table 5:** Antimicrobial treatment patterns.

Treatment Characteristic	Total (n = 33)
**Number of antibiotics per patient, median (IQR)**	3 (2–4)
**Cephalosporins, n (%)**	26 (78.8%)
**Aminoglycosides, n (%)**	19 (57.6%)
**Beta-lactam/beta-lactamase inhibitor, n (%)**	14 (42.4%)
**Vancomycin or linezolid, n (%)**	8 (24.2%)
**Antifungal therapy, n (%)**	6 (18.2%)
**Dual Gram-positive and Gram-negative coverage, n (%)**	30 (90.9%)

**Table 6:** Factors associated with relapsing peritonitis.

Variable	Relapse (n = 4)	No Relapse (n = 29)	*p*-Value
**Age, years, median [IQR]**	66 (58–77)	57 (43–72)	0.42 ^1^
**Female sex, n (%)**	1 (25.0)	15 (51.7)	0.61 ^2^
**Diabetes mellitus, n (%)**	3 (75.0)	9 (31.0)	0.12 ^2^
**Hemoglobin, g/dL, median (IQR)**	9.6 (8.5–10.4)	11,2 (10.9–12.1)	0.08 ^1^
**Gram-negative etiology, n (%)**	3 (75.0)	13 (44.8)	0.34 ^2^
**≥3 antibiotics used, n (%)**	4 (100)	10 (34.5)	0.02 ^2^

Note: Data are presented as median (IQR) or n (%). ^1^ Mann–Whitney U test; ^2^ Fisher’s exact test. IQR: interquartile range.

## Data Availability

The data presented in this study are available upon request from the corresponding author. The data are not publicly available due to patient confidentiality.
